# Human social conditions predict the risk of exposure to zoonotic parasites in companion animals in East and Southeast Asia

**DOI:** 10.1038/s43856-022-00210-8

**Published:** 2022-11-15

**Authors:** Vito Colella, Phrutsamon Wongnak, Yi-Lun Tsai, Viet-Linh Nguyen, D. Y. Tan, Kenneth B. Y. Tong, Na Lu, Fang Fang, Yin Zhijuan, Jiangwei Wang, Xin Liu, Junyan Dong, Wisnu Nurcahyo, Upik K. Hadi, Virginia Venturina, Piyanan Taweethavonsawat, Saruda Tiwananthagorn, Thong Q. Le, Khanh L. Bui, Malaika Watanabe, Puteri A. M. A. Rani, Rebecca J. Traub, Frédéric Beugnet, Karine Chalvet-Monfray, Lénaïg Halos

**Affiliations:** 1 University of Melbourne, Melbourne, VIC Australia; 2 University of Bari, Bari, Italy; 3 Université de Lyon, Lyon, France; 4 Université Clermont Auvergne, Clermont-Ferrand, France; 5 National Pingtung University of Science and Technology, Pingtung, Taiwan; 6 Boehringer Ingelheim Animal Health, Lyon, France; 7 Animal & Avian Veterinary Clinic, Yishun, Singapore; 8 College of Animal Science and Technology, Guangxi University, Nanning, China; 9 Sapphire Veterinary Hospital, Shanghai, China; 10 Meilian Zhonghua Veterinary Referral Center, Beijing, China; 11 Nanjing Police Dog Research Institute, Nanjing, China; 12 Gadjah Mada University, Yogyakata, Indonesia; 13 IPB University Indonesia, Bogor, Indonesia; 14 Central Luzon State University, Muñoz, Philippines; 15 Chulalongkorn University, Bangkok, Thailand; 16 Chiang Mai University, Chiang Mai, Thailand; 17 Nong Lam University, Ho Chi Minh City, Vietnam; 18 Vietnam National University of Agriculture, Hanoi, Vietnam; 19 University Putra Malaysia, Seri Kembangan, Malaysia

**Keywords:** Parasitic infection, Epidemiology

## Abstract

**Background:**

A recent dramatic surge in pet ownership has been observed across metropolitan areas in Asia. To date, there is a dearth of information on the risk associated with pet ownership for the transmission of parasites on a large scale in Asia, despite this continent giving rise to the largest burden of zoonotic infections worldwide.

**Methods:**

We explored the nature and extent of zoonotic internal (endo-) and external (ecto-) parasites and arthropod-borne pathogens in 2381 client-owned dogs and cats living in metropolitan areas of eight countries in East and Southeast Asia using reliable diagnostic tests and then undertook extensive statistical analyses to define predictors of exposure to zoonotic pathogens.

**Results:**

The estimated ORs for overall parasite infections are 1.35 [95% CIs 1.07;1.71] in young animals and 4.10 [1.50;11.2] in the animal group older than 15 years as compared with adult animals, 0.61 [0.48;0.77] in neutered animals as compared to unneutered animals, 0.36 [0.26;0.50] in animals living in urban areas as compared with rural areas, 1.14 [1.08;1.21] for each 1 °C increase of annual mean temperature which varies from 12.0 to 28.0 °C, and 0.86 [0.78;0.95] for each year of life expectancy which varies from 70.9 to 83.3 years.

**Conclusions:**

Here we highlight the influence of human life expectancy and the neutering status of the animals, which reflect increased living standards through access to education and human and veterinary health care, to be both strongly associated with exposure to zoonotic parasites. An integrated approach of local and international authorities to implement and manage educational programs will be crucial for the control of zoonotic infections of companion animals in Asia.

## Plain language summary

Parasites live on or inside animals or humans and can cause disease. Companion animals (pets) with parasites present a potential risk to the health of their owners, as certain kinds of parasites (known as zoonotic parasites) can affect both animal and human health. Here, we investigated whether human social conditions are associated with zoonotic parasite infections in companion animals in East and Southeast Asia. We found that higher human life expectancy and neutering of the companion animals were associated with fewer zoonotic parasite infections in the animals. These findings highlight the need for an enhanced commitment of local authorities to establish prevention campaigns, including education programs, against zoonotic pathogens. These measures will play a crucial role in alleviating the impact of these diseases in companion animals and humans in Asia.

## Abstract (web summary)

Colella, Wongnak et al. test pet dogs and cats from metropolitan areas of eight countries in East and Southeast Asia for zoonotic parasites. The authors identify factors associated with potential exposure to zoonotic parasites, including animal characteristics and human living conditions.

## Introduction

Asia is one of the largest and most densely populated continents, home to more than half of the world’s human population. In the last two decades, many Asian countries, such as Taiwan, Malaysia, Singapore, and China, have undergone massive socioeconomic and urban transitions, leading to a substantial increase in individual wealth and living standards^1^. Nonetheless, such an increase in living conditions is not unified across the continent with wealth unevenly distributed between and within Asian countries. This inequality is well reflected in life expectancy and diseases, with people from disadvantaged countries being most adversely and chronically impacted^2^. Infectious diseases, particularly those caused by zoonotic and neglected parasites (including protists, helminths, and arthropods), represent a major and long-term burden in disadvantaged communities^2^; many of these pathogens are carried by animals, such as dogs and cats, and include fleas (and the pathogens they transmit) and soil-transmitted helminths, which can cause chronic and/or serious illnesses in infected people^2–9^. The status of dogs and cats kept as companion animals in Asia is a relatively recent phenomenon and is highly correlated with a modern urban lifestyle. However, while some parasites are well-recognized causes of diseases in people from Asia, almost nothing is known about the prevalence, distribution, and predictors for exposure to zoonotic parasites in the reservoir populations living in metropolitan areas. Nonetheless, in advantaged urban areas in Asia, the number of dogs and cats has surged as a consequence of people’s wealth (particularly over the last decade) and continues to grow at a major rate^10^. Hence, despite the undisputable benefits of the human–animal bond^11^, the risk of acquiring zoonotic pathogens from companion animals is a real threat in these regions^9,12^.

Although the risk indicators associated with this zoonotic transmission are assumed, they can be challenging to quantitate; epidemiological information for most communities is often missing, and, importantly, the predictors for exposure to zoonotic pathogens have not yet been established in Asia. Here, we hypothesize that, together with animal characteristics and bioclimatic factors, people’s standard of living can predict the risk of exposure to zoonotic parasites in companion animals sharing the same metropolitan areas in Asia as one of their owners. To test this hypothesis, we explored here the nature and extent of zoonotic internal (endo-) and external (ecto-) parasites and arthropod-borne zoonotic pathogens in more than 2300 client-owned dogs and cats living in metropolitan areas of eight countries in East and Southeast Asia using a panel of reliable diagnostic tests and then undertook extensive statistical analyses to define predictors of exposure to zoonotic pathogens. Here we highlight the influence of human life expectancy and the neutering status of the animals, which reflect increased living standards through access to education and human and veterinary health care, to be both strongly associated with exposure to zoonotic parasites

## Methods

### Study sites and sample collection

Academic institutions and private facilities from EA (China and Taiwan) and SEA (Indonesia, Malaysia, Philippines, Singapore, Thailand, and Viet Nam) collaborated on the study. The in-life phase lasted from June 2017 to July 2018. On a monthly basis, we sampled 10 client-owned dogs and 10 client-owned cats in Taiwan, Indonesia, Malaysia, the Philippines, Singapore, Thailand, and Vietnam and 40 dogs and 40 cats in Mainland China for a target total sample size of 2640 companion animals (Supplementary Table 1).

All the animals enrolled in this study were recruited by veterinarians working in academic institutions or private facilities for routine procedures, e.g., annual check-ups and vaccination. The inclusion criteria were: (1) A history of regular access to outdoor environments; (2) and having not received recent antiparasitic treatments (~2 weeks). Owners of the animals were asked to read, accept, and sign a consent form, containing the study protocols described in each country’s official language before recruiting their animals into this study. Once included, each animal was identified with a unique identification serial number. The Ethics Committee of the Department of Veterinary Medicine, University of Bari approved the protocol of this study (protocol no. 13/17). At inclusion, we completed a questionnaire on background information on each animal (age, sex, husbandry, household type, clinical status, neuter status) before performing a complete check-up including clinical evaluation, ectoparasite collection, blood sampling, and feces collection. We conducted specific on-site training sessions in China, Vietnam, Thailand, and Indonesia, and online for Malaysia, Singapore, the Philippines, and Taiwan to guarantee compliance with the procedures of the study protocol.

### Parasite detection and identification

Methods and data on the detection and identification of ectoparasites and vector-borne pathogens are reported in Colella et al. (2020)^9^. For endoparasites, we collected freshly voided feces from each animal and kept them refrigerated before processing within 24 h. Subsequently, the occurrence of endoparasites in fecal samples was detected by a flotation technique with a solution with a specific gravity of 1.200–1.350 (zinc sulfate or sodium chloride), a sedimentation technique, and a Baermann–Wetzel technique following standardized methods^13^. To assess the occurrence of the zoonotic *A. ceylanicum*, and detect species of hookworms infecting pets in Asia, for any samples that yielded a positive result for nematode eggs in the family Ancylostomatidae, the supernatant of the floatation solution, and/or an aliquot of the Baermann–Wetzel examination, and/or an aliquot of the sedimentation solution were transferred into tubes containing 70% ethanol, then analyzed at the University of Melbourne, Australia through a multiplex qPCR assay for species identification^14^. While the identification of other endoparasite groups was supported by morphological characteristics under microscopic observations, and therefore it was conducted at a family/genus level rather than a species level.

### Risk indicator variables

We compiled a set of potential risk indicators of the exposure to ectoparasites (fleas, ticks, mites and/or lice), endoparasites (Toxocaridae, Ancylostomatidae, Coccidia, fluke, *Trichuris* sp., *Strongyloides* sp., *Dipylidium* sp., Diphyllobothriidae, and/or Trichomonads), vector-borne pathogens (VBP) (*Anaplasma* spp., *Ehrlichia* spp., *Borrelia burgdorferi* sensu lato, *Leishmania infantum, Dirofilaria immitis* and/or Apicomplexan), and overall parasites (ectoparasites, endoparasites, and/or VBP). We choose to keep the general terminology parasites even if it includes three bacterial VBP. Four groups of risk indicators were considered: (1) Animal characteristics (species, age class, sex, neuter status); (2) Husbandry (rural or urban environment and household type, with or without garden); (3) Bioclimatic factors (annual mean temperature and annual precipitation); (4) Socio-economic factors (human population density, pet-human population ratio, and human life expectancy). The source of information, the unit, the type of variables, and a brief description of each variable are provided in Table 1. These variables were selected based on common knowledge of the factors influencing host–parasite interaction.

**Table 1:** List of potential risk indicators for parasite infection status used in regression analysis and related variables.

Group	Variable name	Type	Description	Source/reference
Animal characteristics	Animal species	Categorical	2 categories (Cat; Dog)	Animals
	Age class	Categorical	3 categories (<5 year; 5–15 years; >15 years)	Animals
	Sex	Categorical	2 categories (Male; Female)	Animals
	Neuter	Categorical	2 categories (Yes; No)	Animals
Bioclimatic factors	Annual mean temperature	Continuous	The annual mean of monthly average temperature indicated by city (in °C; BIO 1)	Worldclim database^16^
	Annual precipitation	Continuous	The sum of all total monthly precipitation indicated by city (in mm; BIO 12)	Worldclim database^16^
Husbandry	Environment	Categorical	2 categories (Rural area: mainly vegetated environment; Urban area: mainly surrounded by buildings)	Animals
	Household	Categorical	2 categories (Apartment; House with garden)	Animals
Socio-economic factors	Human population density	Continuous	Human population in 2019 density indicated by city (in estimated numbers of individuals/km^2^)	World pop data^17^
	Human population	Continuous	Human population in 2019 indicated per country/territory (in estimated individual counts)	World Bank data^17^
	Cat population	Continuous	Population of cats per country/territory in 2018 (in estimated individual counts)	The Boehringer-Ingelheim Animal Health animal census 2018
	Dog population	Continuous	Population of dogs per country/territory in 2018 (in estimated individual counts)	The Boehringer-Ingelheim Animal Health animal census 2018
	Pet–human population ratio	Continuous	A ratio between pet (cats and dogs) population and human population indicated by country/territory	Calculated from cat, dog, and human populations
	Human life expectancy	Continuous	Life expectancy of the population at birth in 2017, indicated by country/territory (in years)	World Bank data^18^

The source of information, the unit, the type of variables, and a brief description of each variable are provided.

Animal characteristics and environment/management data were recorded by local investigators. Animal age was classified into three groups as (1) <5 years; (2) between 5 and 15 years; (3) more than 15 years. The environment was defined by the features of the surrounding environment as rural area (mainly vegetated) or urban (mainly buildings), while household types were characterized as an apartment (without a garden) or farm/house with a garden. The annual mean temperature (in °C) and the annual precipitation (in mm of precipitation) of each city were derived from the WorldClim database^15^ (BIO 1 and BIO 12, respectively). The city-level human population density (individuals/km^2^) was estimated from the WorldPop data^16^. The pet-human population ratio for each country was calculated from the sum of dog and cat populations in 2018 acquired from the animal consensus of the Boehringer Ingelheim Animal Health, and the human population data in 2019 from the World Bank data^17^. Finally, the country-level life expectancy of the human population at birth in 2017 was acquired from the World Bank data^18^.

### Statistical analyses

All the statistical analyses were carried out using R programming language version 3.6.0^19^. Descriptive analyses were carried out to characterize the demographics and the infection rate of dog and cat populations. Associations among parasite infection status and potential risk indicators were assessed by Cramér’s V statistics and a multiple correspondence analysis (MCA), treating all variables as categorical variables, using sjstats^20^ and ade4^21^ packages, respectively. The risk indicators were subsequently identified by a multivariable mixed-effects logistic regression shown in Eq. (1) using *lme4* package^22^. Animals with missing data were disregarded in the multivariate analysis.

1 $${{{{{\rm{logit}}}}}}[{{\Pr }}({y}_{i}\,j=1)]={\beta }_{0}+{\sum }_{k=1}^{n}{\beta }_{k}{X}_{ij}+{u}_{j}+{\varepsilon }_{ij}$$

The infection status *y*_*ij*_ of an individual *i* was used as the response variable, while the city *j* was used to indicate the random effect. *y*_*ij*_ was assumed to follow a binomial distribution, where *y*_*ij*_ = 1 indicated that the animal was infected. *β*_0_ is the fixed intercept, *β*_*k*_ is the fixed effects, *n* is the total number of fixed effects, and *X*_*ij*_ is the covariates included in the model. *u*_*j*_ represents the random effects on the intercept and *ε*_*ij*_ is the unstructured error, both were assumed normally distributed. A multivariable model of the fixed effects was generated by both forward and backward selection of significant variables decreasing the Akaike information criterion (AIC). The significance level of 10% was used to reject the models. A correlation matrix of all pairwise combinations of variables was carried out to assess the collinearity. Area under curve (AUC) was calculated for receiver operating characteristic (ROC) curves to assess the predictive ability of the models. The selection of the best models was based on the following criteria: (1) minimal AIC and (2) maximal AUC for the ROC curve. Besides, we also carried out a posteriori verification of the models by testing the sensitivity of the parameters to extreme values.

### Reporting summary

Further information on research design is available in the Nature Research Reporting Summary linked to this article.

## Results

### Demographic characteristics

A total of 2381 client-owned animals, including 1229 dogs (565 females, 660 males, and 4 unreported) and 1152 cats (543 females, 606 males, and 3 unreported), were included in this study. Overall, 65.8% (1713/2381) of these animals were <5 years of age, with the median age, 5th and 95th percentiles of 2, 0.3, and 12 years, respectively. Most (85.8%, 2043/2381) of these animals lived in highly urbanized areas, and 66% (1571/2381) lived in a house with access to a garden. In total, 35.8% (854/2381) of the animals had been reported to be neutered. The recruited dog population was older and less neutered than the cat population (Table 2).

**Table 2:** Reported demographic characteristics of recruited client-owned dog and cat populations.

Demographic characteristics	Cats and dogs^a^ (*n* = 2381) (%)		Cats^a^ (*n* = 1152) (%)		Dogs^a^ (*n* = 1229) (%)		*p*-value^b^
*Age (years)*							<0.001
<5	65.8	(1566)	79.9	(921)	52.5	(645)	
5–15	32.0	(763)	18.1	(208)	45.2	(555)	
>15	0.83	(20)	0.61	(7)	1.06	(13)	
*Sex*							NS
Male	53.2	(1266)	52.6	(606)	53.7	(660)	
Female	46.5	(1108)	47.1	(543)	46.0	(565)	
*Neuter*							<0.001
Yes	35.9	(854)	43.1	(496)	29.1	(358)	
No	58.4	(1390)	48.7	(561)	67.5	(829)	
*Environment*							NS
Urban area	85.8	(2043)	87.2	(1004)	84.5	(1039)	
Rural area	14.2	(337)	12.8	(147)	15.5	(190)	
*Household*							NS
Apartment	34.0	(809)	34.5	(398)	33.4	(411)	
House with garden	66.0	(1571)	65.4	(753)	66.6	(818)	

^a^The results were reported as a percentage (number of animals).

^b^*p*-value for the *χ*^2^ test/Fisher’s exact test for the differences between dog and cat populations, NS indicated a non-significant difference with a *p*-value ≥ 0.05.

### Occurrence of parasites in companion animals

**Fig. 1:**
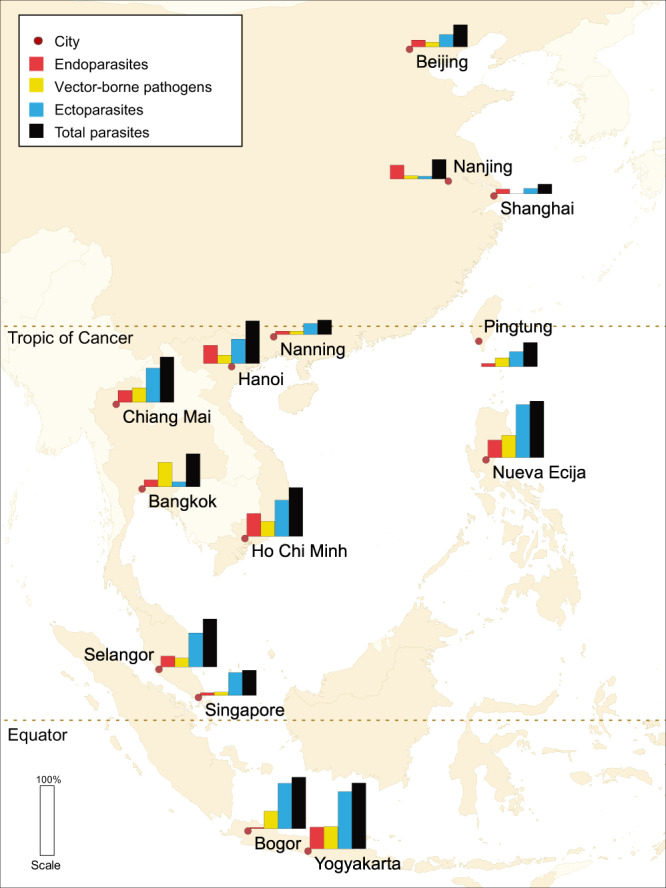
Geographical distribution of parasites detected in client-owned animals (dogs and cats) in 14 cities of East and South-East Asia. Ectoparasites (fleas, ticks, mites and/or lice), Endoparasites (Toxocaridae, Ancylostomatidae, *Trichuris* sp., *Strongyloides* sp., *Dipylidium* sp., Diphyllobothriidae, Trichomonads, Coccidia, and flukes), Vector-borne pathogens (*Anaplasma* spp., *Ehrlichia* spp., *Borrelia burgdorferi* sensu lato, *Leishmania infantum*, *Dirofilaria immitis* and/or Apicomplexa). The map was created using QGIS version 3.8, Zanzibar (https://www.qgis.org).

**Fig. 2:**
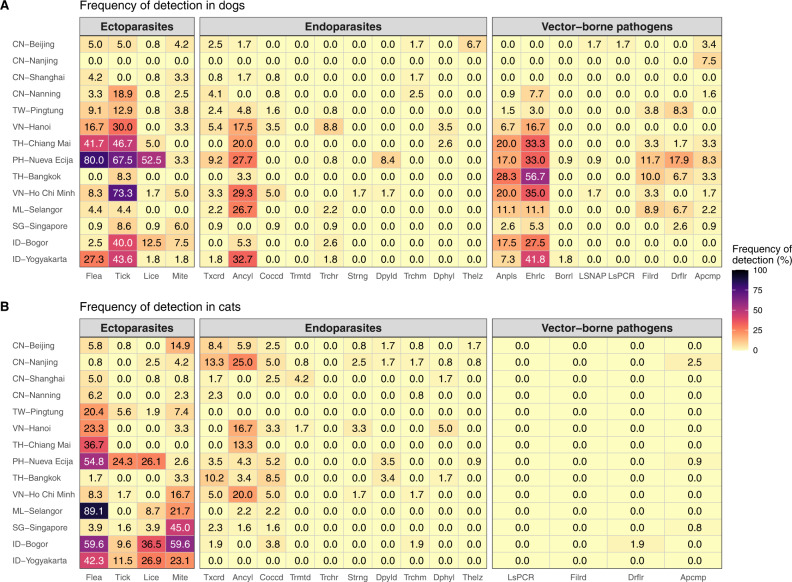
Frequency of detection of parasites in companion animals (dogs and cats) in East and Southeast Asia, classified by city and type of parasite: **A** Dogs and **B** Cats. Country names and parasite types are abbreviated as CN Mainland China, TW Taiwan, VN Viet Nam, TH Thailand, PH Philippines, ML Malaysia, SG Singapore, ID Indonesia, Anpls *Anaplasma* spp., Ehrlc *Ehrlichia* spp., Borrl *Borrelia* spp., LSNAP *Leishmania infantum* (detected by SNAP), LsPCR *Leishmania infantum* (detected by PCR), Filrd Filarial parasites, Drflr *Dirofilaria immitis*, Apcmp Apicomplexans, Txcrd Toxocaridae, Ancyl Ancylostomatidae, Coccd *Coccidia*, Trmtd Trematode, Trchr *Trichuris* spp., Strng *Strongyloides* spp., Dpyld *Dipylidium* spp., Trchm Trichomonads, Dphyl Diphyllobothriidae, Thelz *Thelazia callipaeda.*

Of the enrolled animals, 44.9% [95% confidence interval: 42.9%; 47.0%] were diagnosed to harbor at least one parasite. The occurrence of ectoparasites, VBPs, and endoparasites recorded in all recruited animals was 31.4% [29.6%; 33.3%], 13.1% [11.7%; 14.5%], and 13.5% [12.1%; 14.9%], respectively. The percentages of dogs and cats with one or more parasites were similar, except for VBPs, which were more abundant in dogs (Table 3). The highest percentage of animals recorded to be infected was in Yogyakarta, Indonesia 94.4% [86.2%; 98.4%], followed by 81.4% [75.8%; 86.2%] in Nueva Ecija, the Philippines, and 73.7% [63.9%; 82.1%] in Bogor, Indonesia, while least were affected in China: 14.2% [10.0%; 19.2%] in Shanghai, 20.7% [15.8%; 26.3%] in Nanning, and 27.5% [22.0%; 33.6%] in Nanjing (Figs. 1, 2, Supplementary Fig. 1 and Supplementary Table 2). Morphological and molecular data on the species of ectoparasites and VBPs by country have been reported by Colella et al. (2020)^9^.

**Table 3:** Occurrence of ectoparasites, vector-borne pathogens and endoparasites in client-owned dogs and cats from Eastern and Southeast Asia.

Parasite	Cats^a^	Dogs^a^
Ectoparasites	31.0 [28.3; 33.7]	31.8 [29.2; 34.5]
Fleas	19.6 [17.4; 22.0]	14.8 [12.9; 16.9]
Ticks	3.73 [2.71; 5.00]	22.3 [20.0; 24.7]
Mites	13.3 [11.4; 15.4]	3.01 [2.13; 4.13]
Lice	6.08 [4.77; 7.62]	6.35 [5.05; 7.86]
Vector-borne pathogens	0.93 [0.45; 1.71]	23.8 [21.4; 26.3]
*Anaplasma* spp*.*^b^	–	7.08 [5.71; 8.68]
*Ehrlichia* spp*.*^b^	–	14.8 [12.9; 17.0]
*Borrelia burgdorferi* sensu lato^b^	–	0.16 [0.02; 0.60]
*Leishmania infantum* (antibody)	–	0.33 [0.09; 0.84]
*Leishmania infantum* (PCR)	0.00 [0.00; 0.34]	0.16 [0.02; 0.59]
Filarial parasites (PCR)	0.00 [0.00; 0.34]	2.69 [1.86; 3.75]
*Dirofilaria immitis* (antigens)	0.09 [0.00; 0.50]	3.46 [2.50; 4.65]
Apicomplexan	0.45 [0.15; 1.06]	2.60 [1.79; 3.66]
Endoparasites	14.1 [12.1; 16.3]	12.9 [11.0; 14.9]
Toxocaridae	4.18 [3.10; 5.50]	2.60 [1.77; 3.67]
Ancylostomatidae	6.70 [5.32; 8.30]	9.38 [7.79; 11.2]
Coccidia	2.87 [1.99; 4.01]	0.84 [0.40; 1.53]
Trematode	0.61 [0.25; 1.25]	0.00 [0.00; 0.31]
*Trichuris* sp.	0.00 [0.00; 0.32]	0.92 [0.46; 1.64]
*Strongyloides* spp.	0.61 [0.25; 1.25]	0.08 [0.002; 0.47]
*Dipylidium* sp.	0.87 [0.42; 1.59]	0.92 [0.46; 1.64]
Trichomonads	0.52 [0.19; 1.13]	0.57 [0.23; 1.18]
Diphyllobothriidae	0.61 [0.25; 1.25]	0.25 [0.05; 0.73]
Eyeworm (*Thelazia callipaeda*)	0.35 [0.09; 0.89]	0.65 [0.28; 1.28]
All parasites	43.2 [40.3; 46.2]	46.5 [43.7; 49.4]

^a^The results were reported as a percentage [95% confidence interval for binomial proportion].

^b^Bacterial vector-borne pathogens.

Using conventional coprodiagnostic methods, the most common endoparasites detected in dogs and cats were blood-feeding hookworms (family Ancylostomatidae) (Table 3), recovered from 8.06% [7.00; 9.24%] of all animals, followed by ascaridoid nematodes, found in 3.37% [2.68; 4.18%], and coccidians in 1.83% [1.33; 2.46%], respectively. Trematode eggs were identified in fecal samples from seven cats from Shanghai, Nanjing, and Hanoi, while diphyllobothriid (cestode) eggs were detected in three dogs from Vietnam (Hanoi) and Thailand (Chiang Mai) and in seven cats from the same locations, in addition to Bangkok, Thailand as well as Nanning and Shanghai in China. *Dipylidium* egg packets were detected in 11 dogs from the Philippines (Nueva Ecija) and Vietnam (Hanoi). *Trichuris* sp. was recovered from ten dogs in Vietnam (Hanoi), Taiwan, Philippines (Nueva Ecija), Malaysia (Selangor), and Indonesia (Bogor and Yogyakarta). Trichomonads trophozoites were identified in the feces of seven Chinese dogs from Shanghai, Nanning, and Beijing. Eyeworms, morphologically identified as *Thelazia callipaeda*, were collected from four cats in China (Beijing and Nanjing), and from eight dogs in China (Beijing).

Using a molecular diagnostic approach (qPCR), the hookworms detected by conventional coprodiagnosis (described above) were identified as *Ancylostoma caninum*/*Ancylostoma tubaeforme*, *Ancylostoma ceylanicum*, *Ancylostoma braziliense* and *Uncinaria stenocephala* in 52.6%, 26.3%, 15.8%, and 5.3%, in dogs, and 38.9%, 38.9%, 17.1%, and 5.1% in cats, respectively. *Ancylostoma braziliense* hookworms were detected in cats from Taiwan and Thailand, and in dogs from Taiwan and Vietnam.

### Assessment of risk indicators

Details of risk indicators, including animal characteristics (species, age class, sex, neutered status), animal husbandry (environment and household types), bioclimatic factors (annual mean temperature and annual precipitation), and socio-economic factors (human population density, pet-human population ratio, and human life expectancy) used in the risk factor analyses are reported in Table 1 and Supplementary Table 3. We focused on the three main categories of parasites— ectoparasites, endoparasites and VBPs together (all parasites) and separately, and on specific groups of parasites of public health importance (Ancylostomatidae, ticks, and fleas) for subsequent analyses.

**Fig. 3:**
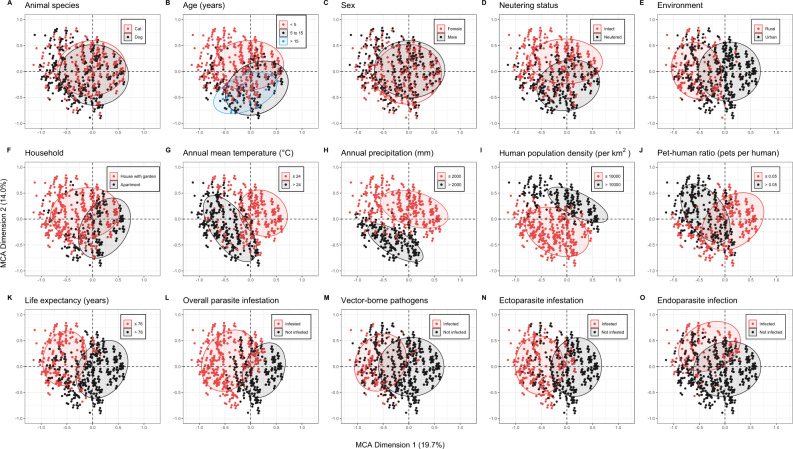
Multiple correspondence analysis of potential risk indicators for parasite, vector-borne pathogens, ectoparasite, and endoparasite infections of individual animals. Points represent individual animals projected on the first two MCA dimensions that explained most variations of characteristics of all animals. The MCA Dimension 1 (horizontal axis) explained 19.7% of total variations while the MCA Dimension 2 (vertical axis) explained 14.0% of total variations. The colors of each potential risk factor indicate different subgroups, as indicated in their legends of animal species **A** age **B** sex **C** neutering status **D** environment **E** household **F** annual mean temperature **G** annual precipitation **H** human population density **I** pet-human ratio **J** life expectancy **K** for overall parasite infestation **L** vector-borne pathogens **M** ectoparasite infestation **N** and endoparasite infection **O**. The eclipses were drawn around the centroid of each subgroup. Potential risk factors and infection statuses that differentiate the animals following the same MCA dimension are considered to be related.

Pairwise and global associations among the parasite infection status in dog and cat populations and potential risk indicators were displayed by Cramér’s V statistics (Supplementary Fig. 2) and multiple correspondence analysis (MCA) (Fig. 3), respectively. The first two dimensions of the MCA explained 33.7% of the variance within the dataset. The first dimension predominantly differentiated individual animals by socio-economic factors (30.3%), followed by bioclimatic factors (19.0%), and husbandry (12.3%). In comparison, the second dimension was driven by bioclimatic factors (32.1%), animal characteristics (29.0%), and socio-economic factors (24.4%). The infection statuses for overall parasites, VBPs, and ectoparasites were distinguished by the first dimension, while endoparasite infection status was mainly involved in the second dimension. Both approaches identified strong correlations among some variables, e.g., the annual mean temperature and the annual precipitation exhibited a strong positive correlation (correlation coefficient = 0.7034 [0.6826; 0.7231]; *p*-value < 0.001), therefore adding both variables in the same regression model does not provide supplementary information.

Odds ratios (ORs) for the infection status of each parasite group are shown in Table 4. The mixed-effects logistic regression models indicated that the overall parasite infection status was associated with all categories of risk indicators (animal characteristics, bioclimatic, husbandry, and socio-economic factors). The estimated ORs for overall parasite infections were 1.35 [1.07; 1.71] in young animals (<5 years) and 4.10 [1.50; 11.2] in the animal group older than 15 years as compared with adult animals (5 to 15 years), 0.61 [0.48; 0.77] in neutered animals as compared to unneutered animals, 0.36 [0.26; 0.50] in animals living in urban areas as compared with rural areas, 1.14 [1.08; 1.21] for each 1 °C of annual mean temperature which varies from 12.0 to 28.0 °C, and 0.86 [0.78; 0.95] for each year of life expectancy which varies from 70.9 to 83.3 years. While the pet-to-human ratio and human population density did not explain the likelihood of parasite exposure.

**Table 4:** Odds ratio (OR) and 95% confidence intervals for the risk indicators associated with exposure to parasites in dogs and cats.

Variable	All parasites	Vector-borne pathogens	Ectoparasites	Endoparasites
Species (dog)	–	34.1 [17.7; 65.8]	–	–
Sex (male)	–	–	–	–
Age class (<5 years)	1.35 [1.07; 1.71]	–	–	2.12 [1.43; 3.15]
Age class (>15 years)	4.10 [1.50; 11.2]	–	–	2.45 [0.52; 11.4]
Neuter (yes)	0.61 [0.48; 0.77]	–	0.53 [0.41; 0.69]	–
Environment (urban area)	0.36 [0.26; 0.50]	–	0.28 [0.21; 0.39]	–
Household (garden)	–	2.49 [1.54; 4.03]	–	–
Average temperature (°C)	1.14 [1.08; 1.21]	1.16 [1.06; 1.26]	– ^a^	–
Average precipitation (mm)	– ^b^	– ^b^	1.001 [1.0006; 1.0014]	–
Human life expectancy (Year)	0.86 [0.78; 0.95]	0.86 [0.76; 0.98]	0.86 [0.73; 0.99]	0.89 [0.78; 1.01]^c^
Pet-Human ratio	–	–	–	–
Population density (per km^2^)	–	–	–	–
Variance (City)	0.2769	0.4777	1.023	0.7102
Area under ROC curve	0.7826	0.6585	0.8188	0.7375

References for qualitative variables are, respectively, for species (cat), sex (female), age class (5–15 years), neuter (no), environment (rural area), household (apartment). For quantitative variables, the OR corresponds to an increase of 1 unit.

^a^Bioclimatic variables show a strong association; therefore when the average precipitation effect is taken into account, the average temperature effect is not significant.

^b^Bioclimatic variables show a strong association; therefore when the average temperature effect is taken into account, the average precipitation effect is not significant.

^c^Significant at 10% level (*p*-value = 0.079).

The VBP exposure status was not strongly correlated with the ectoparasite infestation (Cramér’s *V* statistics = 0.1698). Dogs were more likely to be exposed to VBPs than cats, with an OR of 34.1 [17.7; 65.8]. Animals living in a house with access to a garden, and in a city with higher annual mean temperature (or higher annual precipitation) had higher odds of VBP exposure, whilst animals from a country with a higher human life expectancy were less likely to be VBP positive (Table 4).

For the ectoparasite infestation, OR in neutered animals was 0.53 [0.41; 0.69] as compared with unneutered animals, and an OR in animals from urban areas was 0.28 [0.21; 0.39] as compared with rural areas, while the odds of infestation by ectoparasites was higher in a city with higher annual precipitation (or higher annual mean temperature) (Table 4). Ticks and fleas shared similar environmental/management and bioclimatic risk indicators with the overall ectoparasite infestation (Table 5). Dogs were more likely to be infested by ticks (OR = 11.6 [7.83; 17.3]), while they were less affected by fleas (0.53 [0.39; 0.73]) as compared to cats. Fleas tended to infest animals older than 15 years with an OR of 3.68 [1.07; 12.7] compared to animals between 5 and 15 years, and they were less observed in neutered animals with an OR of 0.64 [0.45; 0.92]. Both flea and tick infestations were less abundant in countries with a higher life expectancy.

**Table 5:** Odds ratio (OR) and 95% confidence intervals (CI) for the risk indicators associated with Ancylostomatidae hookworms, ticks, and fleas in dogs and cats.

Variable	Ancylostomatidae	Ticks	Fleas
Species (dog)	1.65 [1.17; 2.33]	11.6 [7.83; 17.3]	0.53 [0.39; 0.73]
Sex (male)	–	–	–
Age class (<5 years)	2.35 [1.38; 4.02]	–	0.78 [0.53; 1.14]
Age class (>15 years)	5.86 [1.11; 30.9]	–	3.68 [1.07; 12.7]
Neuter (yes)	–	0.69 [0.46; 1.05]^d^	0.64 [0.45; 0.92]
Environment (urban area)	–	0.32 [0.21; 0.47]	0.31 [0.22; 0.44]
Household (garden)	–	–	–
Average temperature (°C)	–	1.25 [1.09; 1.42]	– ^a^
Average precipitation (mm)	–	– ^b^	1.0007 [1.0002; 1.0012]
Human life expectancy (Year)	0.89 [0.78; 0.98]^c^	0.82 [0.68; 0.98]	0.78 [0.64; 0.96]
Pet-Human ratio	–	–	–
Population density (per km^2^)	–	–	–
Variance (City)	1.883	1.092	1.365
Area under ROC curve	0.7985	0.9027	0.8765

References for qualitative variables are respectively for species (cat), sex (female), age class (5–15 years), neuter (no), environment (rural area), household (apartment). For quantitative variables, the OR corresponds to an increase of 1 unit.

^a^Bioclimatic variables show a strong association; therefore when the average precipitation effect is taken into account, the average temperature effect is not significant.

^b^Bioclimatic variables show a strong association; therefore when the average temperature effect is taken into account, the average precipitation effect is not significant.

^c^The finding is significant at the 10% level (*p*-value = 0.067).

^d^The finding is significant at the 10% level (*p*-value = 0.084).

The regression model determined age as the only risk indicator for endoparasite infection, with ORs of 2.12 [1.43; 3.15] in animals younger than 5 years as compared with adult animals (5–15 years) (Table 4). Infections by hookworms were predominantly observed in dogs (OR = 1.65 [1.17; 2.33]) and more frequently detected in young (<5 years) and old animals (more than 15 years), with ORs of 2.35 [1.38; 4.02] and 5.86 [1.11; 30.9] as compared with adult animals, respectively (Table 5). The Akaike information criterion (AIC) and the area under the receiver operating characteristic (ROC) curve for the model selections are reported in Supplementary Tables 4–7.

## Discussion

This international multidisciplinary collaborative project involved veterinary academic institutions as well as private veterinarians and pharmaceutical industry partners across the Asia Pacific and Europe, embodying the concept of One Health. The focus of this investigation was on client-owned dogs and cats because they live in close contact with humans and represent an animal population for which sustainable control strategies can be readily planned and implemented, enabled by sound policies and recommendations.

The present study revealed that almost half of the 2381 animals (dogs and cats) sampled harbored at least one of >40 pathogens detected, with >85% of these animals living in highly urbanized metropolitan areas. We explored the role of factors that would not appear to have a correlation with these pathogens and illustrated how human social conditions may impact parasite transmission and indicated the importance of considering anthropogenic factors in the development of control strategies^23^. Interestingly, here, an increase in people’s life expectancy and the neutering status of an animal were reliable predictors for a substantially low risk of exposure to zoonotic parasites. This finding is interpreted to mirror increased wealth and standard of living for people in urban areas of parts of Asia, allowing them to access human and veterinary health care.

For one year increase in human life expectancy (range 70.9 years in The Philippines to 83.3 in Singapore), we found a 0.86 decrease in the OR for the exposure of pet animals to zoonotic VBPs and ectoparasites. Human life expectancy was selected as a key metric for assessing population health and well-being, rather than gross domestic product, which reflects the economic output of a country and would likely not be an indicator of people’s living standard. Although socio-economic status varies within each country, and we did not assess individual socio-economic factors of pets’ owners, life expectancy represents a summary indicator for the overall health of a population, including access to health services, and was used to perform comparisons between rather than within countries. The strong correlation between neutering and decreased parasite infections indicated enhanced access to veterinary services, including vaccination and parasite control programs. Neutering has been proposed as a strategy for the control of key canine-mediated infections and is also positively associated with changes in human behaviors toward dogs and improved caregiving behaviors^24,25^. For instance, the main factors related to the success in the elimination of rabies (a disease that causes an estimated 59,000 human deaths globally per year) are the control of dog populations and mass anti-rabies vaccination^26^.

Market research^27^ has shown some commonality among pet owners in urban areas across Asian countries in that they (often) represent a demographic of wealthy people who are eager to provide a high standard of care to their pets. The majority of animals enrolled in this study lived in highly urbanized areas and were young animals (<5 years old), which relates well to the recent surge in pet ownership^10^. In doing so, we have excluded animals coming from poorer regions and with less access to veterinary services that may be suffering from higher levels of parasitism than those living in urban areas. These findings may explain the relatively lower prevalence of some parasites herein observed in animals whose owners had access to veterinary care and lived in countries with increased living conditions. Conversely, we enrolled pets that had not received a recent antiparasitic treatment (<2 weeks), and by doing so have potentially excluded a small proportion of animals that would have likely been found parasites-free.

The prevalence of parasites reported in the present study appears to be lower than what would have been expected in free-roaming dogs and cats and can be explained by a high level of companion animal care in urban settings, as described in other geographical regions^28,29^. The low prevalence of protistan parasites detected, particularly *Giardia*, may also be attributable to the (conventional) approach employed for the diagnosis of gastrointestinal parasites, which is more suited for the detection of helminth eggs and may have underestimated the true prevalence of protistan infections^30^. For example, a prior study has reported an infection rate for *Giardia* spp. of >27% in dogs and cats from Chiang Mai in Thailand^31^. In addition, the differences in prevalence data reported between study areas in this study may also be related to the difference in the expertise and experience of technicians in the coprodiagnosis of infections. Nonetheless, the protocol setup, which included a capacity-building component through the provision of technical training at local institutions, aimed to limit the adverse impact of variation in technical competence.

Higher percentages of parasite infections were found in both younger (<5 years old) and older animals (>15 years old), as opposed to those in the age range of 5–15. The influence of host age and the propensity for younger animals to be more at risk of infection is well known^32,33^, but higher rates of infection in older animals are less frequently reported. Hookworms, which were the most frequently recorded zoonotic parasites, were detected in 8% of all animals, with higher prevalence in younger and older dogs compared with animals 5–15 years of age. A similar age pattern has been reported in humans^34^, although recent studies employing molecular methods have revealed that hookworm prevalence steadily increases with age, while the efficacy of drugs follows an inverted trend and decreases in the older human population^35^. With the aid of molecular tools, we identified *A. ceylanicum* in one-quarter of hookworm-positive samples. This is of particular concern, as these infected animals act as reservoir hosts for transmission to humans. While previously considered negligible, *A. ceylanicum* is now the second most commonest hookworm species infecting people in the Asia-Pacific^34^. Similarly, we identified *A. braziliense*, the agent of prolonged creeping eruptions in humans^34^, in Taiwan, where its occurrence was previously unknown. These findings emphasise the need to reassess current strategies for the control of soil-transmitted helminths and elevate the importance of implementing a One Health intervention program in areas where these zoonotic hookworms occur at high prevalence in both human and canine populations^34,35^.

Here we report a comprehensive picture of distribution and risk indicators for a multitude of zoonotic infections in a specific population of urban animals in Asia that may require an adaptive and nuanced parasite control program. Parasites are recognized for their often-complex biology and interactions with their hosts and environments, with several abiotic and/or biotic factors additively, synergistically, or antagonistically influencing parasite transmission^23,36^. This explains the greater parasite species prevalence in dogs and cats living in less urbanized areas.

However, the new place given to the pet animal, as an integral part of the family sharing the living vicinities changes the dynamic of infection, creating new rooms for transmission^37^ and the prevalence observed in the present study highlights the need for education of pet owner and monitoring.

Further, the high proportion of pet dogs and cats found infected with parasites highlights the risk associated with the potential spread of parasites and vectors with relocated/rehomed dogs and cats from Asia to countries where pathogens are not endemic and, vice versa, for traveling animals into endemic countries^38,39^. For instance, a recent outbreak of canine monocytic ehrlichiosis has been discovered in Australia in 2020 where *Ehrlichia canis* had never previously been detected^40^. Comparative genomic analysis of *E. canis* from domestic dogs and tick vectors from Australia suggested that this pathogen may have originated from Asia, and it was rapidly spreading throughout the country^41^. Similarly, limited biosecurity and prevention programs for traveling companion animals, especially dogs, have been indicated for the concerning establishment and spread of the Asian long-horned tick, *Haemaphysalis longicornis*, in the US^42^.

Clearly, the willingness and commitment of owners to provide sound care to their animals should be harnessed to establish tailored prevention programs focused on the reduction in the transmission of zoonotic pathogens. An increase in the number of veterinary medicine curricula and an enhanced commitment of local authorities to establish prevention campaigns against zoonotic pathogens will play a crucial role in alleviating the impact of these diseases on humans. This will necessitate an integrated approach of local and international authorities to implement and manage educational programs, particularly in resource-poor areas, where a negative synergistic effect of limited veterinary education and a low standard of living is expected to foster an increased exposure of people to zoonotic infections.

## Supplementary information

Supplementary Data 1

Supplementary Data 2

Supplementary Information

Description of Additional Supplementary Files

Reporting Summary

## Data Availability

Source data for Figs. 1–3 and for Supplementary Figs. 1, 2 are available in Supplementary Data 1, 2. References for bioclimatic and socioeconomic data are provided in Table 1. The animal population data owned by Boehringer-Ingelheim Animal Health can be made available upon request (frederic.beugnet@boehringer-ingelheim.com). The other original (raw) data can be made available upon request to the corresponding author (vito.colella@unimelb.edu.au).
