# Supplementary material for: Human social conditions predict the risk of exposure to zoonotic parasites in companion animals in East and Southeast Asia
Source: Commun Med (Lond). 2022 Nov 15;2:144. doi: 10.1038/s43856-022-00210-8 (PMC9666534; doi:10.1038/s43856-022-00210-8)

Supporting information

Human social conditions predict the risk of exposure to zoonotic parasites in companion animals in East and Southeast Asia

Vito Colella, Phrutsamon Wongnak, Yi-Lun Tsai, Viet L. Nguyen, Do Y. Tan, Na Lu, Fang Fang, Yin Zhijuan, Jiangwei Wang, Xin Liu, Xinghui Chen, Junyan Dong, Wisnu Nurcahyo, Upik K. Hadi, Virginia Venturina, Kenneth B.Y. Tong, Piyanan Taweethavonsawat, Saruda Tiwananthagorn, Thong Q. Le, Khanh L. Bui, Malaika Watanabe, Puteri A.M.A. Rani, Rebecca J Traub, Frédéric Beugnet, Karine Chalvet-Monfray, Lénaïg Halos

Supplementary Table 1. Number of dogs and cats participated in the study, classified by city.

Country	City	Cats	Dogs	Dogs and Cats
Mainland China	Beijing	121	119	240
	Nanjing	120	120	240
	Nanning	129	122	251
	Shanghai	120	120	240
Taiwan	Pingtung	54	132	186
Indonesia	Bogor	45	57	102
	Yogyakarta	33	38	71
Malaysia	Selangor	46	45	91
Philippines	Nueva Ecija	115	120	235
Singapore	Singapore	129	116	245
Thailand	Bangkok	60	60	120
	Chiang Mai	60	60	120
Vietnam	Hanoi	60	60	120
	Ho Chi Minh	60	60	120
**Total**	**Total**	**1152**	**1229**	**2381**

Supplementary Table 2. Occurrence of pathogens in client-owned animals (dogs and cats) in East and South-East Asia by country and city

Country	City	All parasites	Ectoparasites	Vector-borne pathogens	Endoparasites
Mainland China	Beijing	31.9 [26.1; 38.3]	17.9 [13.3; 23.4]	6.67 [3.86; 10.6]	9.66 [6.23; 14.1]
	Nanjing	27.5 [22.0; 33.6]	3.75 [1.73; 7.00]	5.42 [2.92; 9.08]	20.0 [15.1; 25.6]
	Nanning	20.7 [15.8; 26.3]	15.5 [11.3; 20.6]	4.88 [2.55; 8.37]	4.78 [2.49; 8.20]
	Shanghai	14.2 [10.0; 19.2]	7.50 [4.51; 11.6]	0.00 [0.00; 1.53]	7.08 [4.18; 11.1]
Taiwan	Pingtung	34.8 [27.5; 42.6]	22.0 [16.3; 28.7]	12.7 [7.91; 18.9]	5.03 [2.32; 9.33]
Indonesia	Bogor	73.7 [63.9; 82.1]	64.7 [54.6; 73.9]	24.7 [15.8; 35.5]	2.00 [0.24; 7.04]
	Yogyakarta	94.4 [86.2; 98.4]	81.7 [70.7; 89.9]	32.1 [20.3; 46.0]	31.0 [20.5; 43.1]
Malaysia	Selangor	69.2 [58.7; 78.5]	49.5 [38.8; 60.1]	13.2 [7.00; 21.9]	16.5 [9.53; 25.7]
Philippines	Nueva Ecija	81.4 [75.8; 86.2]	75.7 [69.7; 81.1]	31.8 [25.7; 38.4]	24.8 [19.4; 30.8]
Singapore	Singapore	36.2 [30.2; 42.6]	32.7 [26.8; 38.9]	4.53 [2.28; 7.96]	4.08 [1.97; 7.38]
Thailand	Bangkok	47.1 [37.8; 56.4]	6.67 [2.92; 12.7]	35.0 [26.5; 44.2]	10.1 [5.32; 17.0]
	Chiang Mai	64.9 [55.4; 73.6]	49.2 [39.9; 58.4]	21.2 [14.2; 29.7]	17.0 [10.2; 25.8]
Vietnam	Hanoi	61.2 [51.7; 70.1]	35.0 [26.5; 44.2]	11.8 [6.45; 19.4]	26.5 [18.8; 35.5]
	Ho Chi Minh	70.0 [61.0; 78.0]	51.7 [42.4; 60.9]	22.5 [15.4; 31.0]	33.3 [25.0; 42.5]

Supplementary Table 3. Summary of bioclimatic and socio-economic variables by city

Country/Territory	City	Annual mean temperature (°C)	Annual precipitation (mm)	2019 Human density (per km^2)	2017 Life expectancy (years)	Pet-Human ratio
Mainland China	Beijing	12.0	607	28203.8	76.5	0.0368
	Nanjing	15.6	998	16096.2		
	Nanning	22.3	1381	8366.4		
	Shanghai	16.2	1066	34871.0		
Taiwan	Pingtung	23.8	2743	657.6	80.1	0.1032
Indonesia	Bogor	25.5	3791	7261.2	71.3	0.0100
	Yogyakarta	26.0	2165	5289.3		
Malaysia	Selangor	27.0	2374	4078.0	75.8	0.0370
The Philippines	Nueva Ecija	27.1	1850	1410.8	70.9	0.1626
Singapore	Singapore	27.0	2514	8452.5	83.3	0.0341
Thailand	Bangkok	28.0	1464	15428.0	76.7	0.1634
	Chiang Mai	25.0	1119	1731.5		
Vietnam	Hanoi	23.9	1687	12260.9	75.2	0.0973
	Ho Chi Minh	27.5	1818	23878.1		

Supplementary Table 4. The Akaike information criterion (AIC) and the area under the receiver operating characteristic (ROC) curve for the model selection.

Parasite	Model	Description	AIC	Area under ROC curve
All parasites	A0	Null	2544.08	0.7614
	A1	AgeCl	2534.80	0.7686
	A2	AgeCl + Neut	2519.28	0.7723
	A3	AgeCl + Neut + Envi	2479.83	0.7830
	A4	AgeCl + Neut + Envi + LifeEx	2476.99	0.7828
	**A5**	**AgeCl + Neut + Envi + LifeEx + Temp**	**2467.71**	**0.7826**
	A6	AgeCl + Neut + Envi + LifeEx + Temp + *Prec**	2465.41*	0.7829
	A7	AgeCl + Neut + Envi + LifeEx + Temp + Spec	2469.68	0.7817
	A8	AgeCl + Neut + Envi + LifeEx + Temp + PHratio	2469.69	0.7826
	A9	AgeCl + Neut + Envi + LifeEx + Temp + *House**	2467.62*	0.7827
	A10	AgeCl + Neut + Envi + LifeEx + Temp + Sex	2469.15	0.7834
	A11	AgeCl + Neut + Envi + LifeEx + Temp + PopDens	2469.56	0.7823
Vectorborne pathogens	B0	Null	1428.10	0.7370
	B1	Spec	1166.15	0.6585
	B2	Spec + House	1152.89	0.6582
	B3	Spec + House + LifeEx	1151.43	0.6585
	**B4**	**Spec + House + LifeEx + Temp**	**1144.61**	**0.6585**
	B5	Spec + House + LifeEx + Temp + *PHratio**	1143.64*	0.6536
	B6	Spec + House + LifeEx + Temp + Prec	1146.31	0.6571
	B7	Spec + House + LifeEx + Temp + AgeCl	1145.47	0.6549
	B8	Spec + House + LifeEx + Temp + Neut	1145.57	0.6606
	B9	Spec + House + LifeEx + Temp + Sex	1146.35	0.6592
	B10	Spec + House + LifeEx + Temp + PopDens	1146.44	0.6585

*Indicates the non-significant variables that reduce the AIC value; Bolded rows indicated the selected models for describing risk indicators.

Supplementary Table 5. The Akaike information criterion (AIC) and the area under the receiver operating characteristic (ROC) curve for the model selection (cont.).

Parasite	Model	Description	AIC	Area under ROC curve
Ectoparasites	C0	Null	2224.0	0.79830
	C1	Neut	2205.9	0.80519
	C2	Neut + Envi	2151.3	0.81708
	**C3**	**Neut + Envi + Prec**	**2133.2**	**0.81880**
	C4	Neut + Envi + Prec + Temp	2133.9	0.81838
	C5	Neut + Envi + Prec + *LifeEx**	2131.6*	0.81794
	C6	Neut + Envi + Prec + *AgeCl**	2131.0*	0.81967
	C7	Neut + Envi+ Prec + House	2134.2	0.81831
	C8	Neut + Envi + Prec + PHratio	2134.6	0.81798
	C9	Neut + Envi + Prec + Spec	2134.8	0.81915
	C10	Neut + Envi + Prec + Sex	2135.2	0.81897
	C11	Neut + Envi + Prec + PopDens	2134.7	0.8189
Endoparasites	D0	Null	1549.1	0.72677
	**D1**	**AgeCl**	**1537.6**	**0.73752**
	D2	AgeCl + *LifeEx**	1537.1*	0.73598
	D3	AgeCl + House	1539.5	0.73652
	D4	AgeCl + Envi	1539.4	0.73732
	D5	AgeCl + Spec	1539.3	0.73501
	D6	AgeCl + Neut	1537.7	0.74038
	D7	AgeCl + PHratio	1538.9	0.73708
	D8	AgeCl + Temp	1539.5	0.73749
	D9	AgeCl + Prec	1538.9	0.73705
	D10	AgeCl + *Sex**	1536.1*	0.74157
	D11	AgeCl + PopDens	1539.6	0.73759

*Indicates the non-significant variables that reduce the AIC value; Bolded rows indicated the selected models for describing risk indicators.

Supplementary Table 6. The Akaike information criterion (AIC) and the area under the receiver operating characteristic (ROC) curve for the model selection (cont.).

Parasite	Model	Description	AIC	Area under ROC curve
Ancylostoma sp.	E0	Null	1042.15	0.78878
	E1	AgeCl	1037.59	0.79238
	**E2**	**AgeCl + Spec**	**1031.66**	**0.79849**
	E3	AgeCl + Spec + LifeEx*	1030.84*	0.79736
	E4	AgeCl + Spec + House	1032.08	0.80133
	E5	AgeCl + Spec + Envi	1033.50	0.79832
	E6	AgeCl + Spec + Neut*	1031.53*	0.80209
	E7	AgeCl + Spec + PHratio	1032.24	0.79826
	E8	AgeCl + Spec + Temp	1032.70	0.79858
	E9	AgeCl + Spec + Prec	1033.46	0.79872
	E10	AgeCl + Spec + Sex	1031.79	0.80217
	E11	AgeCl + Spec + PopDens	1032.91	0.79842
Ticks	F0	Null	1377.14	0.83532
	F1	Spec	1175.99	0.89536
	F2	Spec + Envi	1145.93	0.90253
	F3	Spec + Envi + LifeEx	1145.08	0.90066
	**F4**	**Spec + Envi + LifeEx + Temp**	**1138.47**	**0.90270**
	F5	Spec + Envi + LifeEx + Temp + Neut*	1137.50*	0.90167
	F6	Spec + Envi + LifeEx + Temp + Prec	1140.08	0.90293
	F7	Spec + Envi + LifeEx + Temp + PHratio	1139.55	0.90187
	F8	Spec + Envi + LifeEx + Temp + AgeCl	1140.46	0.90291
	F9	Spec + Envi + LifeEx + Temp + Sex	1140.26	0.90219
	F10	Spec + Envi + LifeEx + Temp + House	1140.40	0.90263
	F11	Spec + Envi + LifeEx + Temp + PopDens	1140.44	0.90268

*Indicates the non-significant variables that reduce the AIC value; Bolded rows indicated the selected models for describing risk indicators.

Supplementary Table 7. The Akaike information criterion (AIC) and the area under the receiver operating characteristic (ROC) curve for the model selection (cont.).

Parasite	Model	Description	AIC	Area under ROC curve
Fleas	G0	Null	1411.56	0.86283
	G1	Spec	1400.11	0.86817
	G2	Spec + Envi	1365.60	0.86976
	G3	Spec + Envi + LifeEx	1362.10	0.86942
	G4	Spec + Envi + LifeEx + Neut	1359.43	0.87289
	G5	Spec + Envi + LifeEx + Neut + AgeCl	1357.50	0.87476
	**G6**	**Spec + Envi + LifeEx + Neut + AgeCl + Prec**	**1351.18**	**0.87650**
	G7	Spec + Envi + LifeEx + Neut + AgeCl + Prec + PHratio*	1350.75*	0.87588
	G8	Spec + Envi + LifeEx + Neut + AgeCl + Prec + Temp	1352.73	0.87586
	G9	Spec + Envi + LifeEx + Neut + AgeCl + Prec + Sex	1352.17	0.87634
	G10	Spec + Envi + LifeEx + Neut + AgeCl + Prec + House	1352.52	0.87538
	G11	Spec + Envi + LifeEx + Neut + AgeCl + Prec + PopDens	1351.42	0.87599

*Indicates the non-significant variables that reduce the AIC value; Bolded rows indicated the selected models for describing risk indicators.

Supplementary Figure 1. Geographical distribution of parasites detected in dog (A) and cat (B) populations from 14 cities of East and South-East Asia.

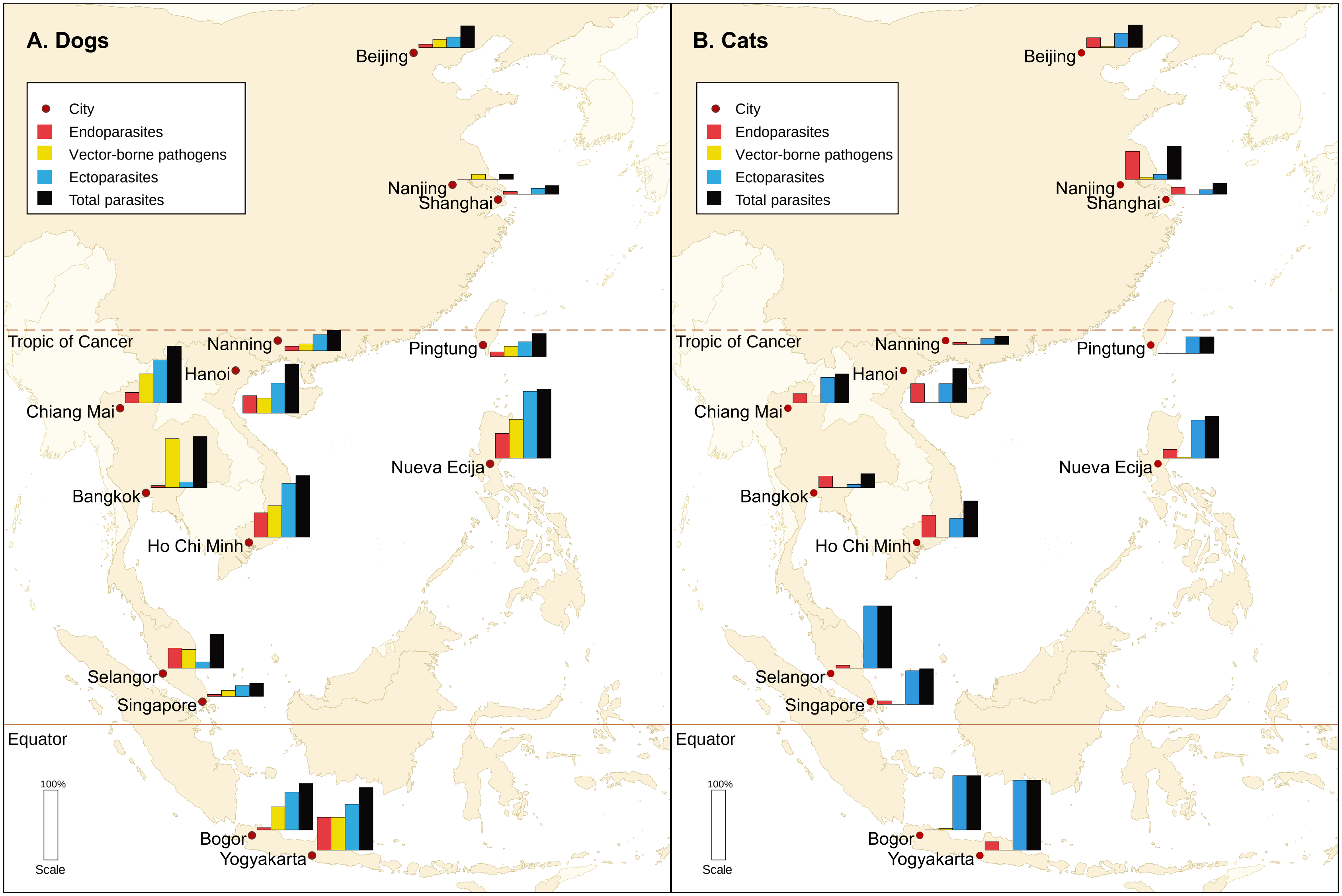

Supplementary Figure 2. Pairwise association between potential risk factors and infection statuses. All variables were treated as categorical variables as: Species (dog and cat); Age (young: < 5 years, adult: 5 to 15 years, old: > 15 years); Sex (M: male, F: female); Neuter (Y: yes, N: no); Environment (RA: rural area, UA: urban area); Average annual temperature (low: ≤ 24 °C, high: > 24 °C); Annual precipitation (low: ≤ 2000 mm, high: > 2000 mm); Population density (low: ≤ 10000 per km^2, > 10000 per km^2); Pet-human ratio (low: ≤ 0.05, high > 0.05); Life expectancy (low: ≤ 76 years, high: > 76 years). Infestation status for all parasites were denoted using 1 as infected and 0 as not infected. Colours represent the value of Cramér's V statistics with asterisks indicating the Cramér's V value greater than 0.3.

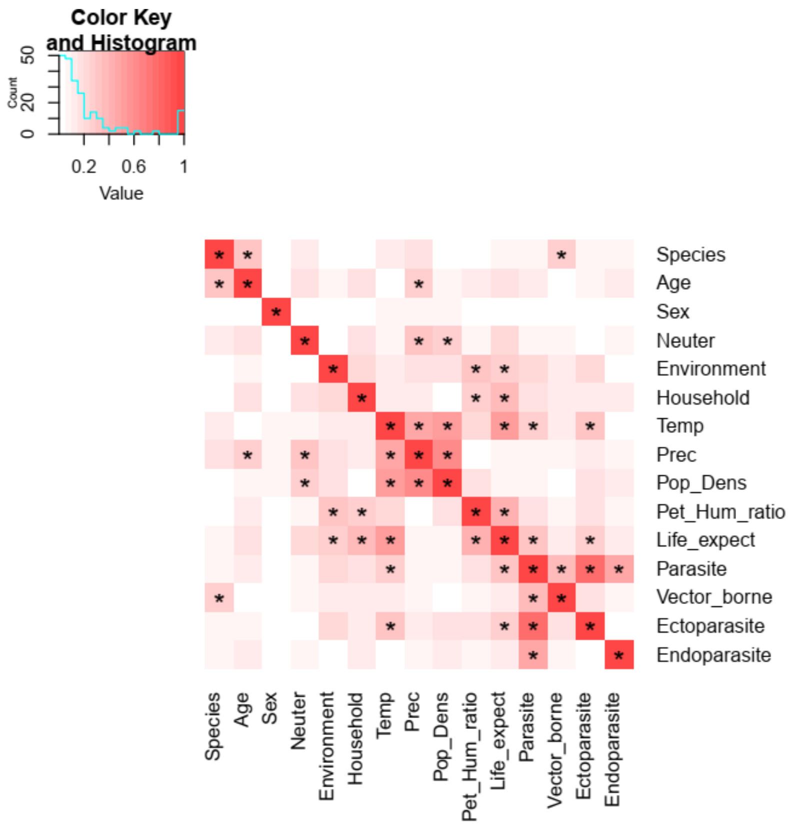

Supplement: Supplementary file 3 — Supplementary Information [file 43856_2022_210_MOESM3_ESM.pdf]
