# Supplementary material for: Human social conditions predict the risk of exposure to zoonotic parasites in companion animals in East and Southeast Asia
Source: Commun Med (Lond). 2022 Nov 15;2:144. doi: 10.1038/s43856-022-00210-8 (PMC9666534; doi:10.1038/s43856-022-00210-8)
Supplement: Supplementary file 4 — Description of Additional Supplementary Files [file 43856_2022_210_MOESM4_ESM.pdf]

Description of Additional Supplementary Files

Supplementary Data 1. Data of ectoparasites, endoparasites, and vector-borne pathogens of dogs and cats in East and Southeast Asia.

Supplementary Data 2. Animal characteristics, bioclimatic, husbandry, and socioeconomic factors related to the infection with ectoparasites, endoparasites, and vector-borne pathogens of dogs and cats in East and Southeast Asia.
