# Supplementary material for: Human social conditions predict the risk of exposure to zoonotic parasites in companion animals in East and Southeast Asia
Source: Commun Med (Lond). 2022 Nov 15;2:144. doi: 10.1038/s43856-022-00210-8 (PMC9666534; doi:10.1038/s43856-022-00210-8)
Supplement: Supplementary file 5 — Reporting Summary [file 43856_2022_210_MOESM5_ESM.pdf]

nature research

Corresponding author(s): Vito Colella

Last updated by author(s): Nov 1, 2022

Reporting Summary

Nature Research wishes to improve the reproducibility of the work that we publish. This form provides structure for consistency and transparency in reporting. For further information on Nature Research policies, see our Editorial Policies and the Editorial Policy Checklist.

Statistics

For all statistical analyses, confirm that the following items are present in the figure legend, table legend, main text, or Methods section.

n/a	Confirmed	
☐	☒	The exact sample size (*n*) for each experimental group/condition, given as a discrete number and unit of measurement
☒	☐	A statement on whether measurements were taken from distinct samples or whether the same sample was measured repeatedly
☐	☒	The statistical test(s) used AND whether they are one- or two-sided<br>*Only common tests should be described solely by name; describe more complex techniques in the Methods section.*
☐	☒	A description of all covariates tested
☐	☒	A description of any assumptions or corrections, such as tests of normality and adjustment for multiple comparisons
☐	☒	A full description of the statistical parameters including central tendency (e.g. means) or other basic estimates (e.g. regression coefficient) AND variation (e.g. standard deviation) or associated estimates of uncertainty (e.g. confidence intervals)
☐	☒	For null hypothesis testing, the test statistic (e.g. *F*, *t*, *r*) with confidence intervals, effect sizes, degrees of freedom and *P* value noted<br>*Give P values as exact values whenever suitable.*
☒	☐	For Bayesian analysis, information on the choice of priors and Markov chain Monte Carlo settings
☒	☐	For hierarchical and complex designs, identification of the appropriate level for tests and full reporting of outcomes
☒	☐	Estimates of effect sizes (e.g. Cohen's *d*, Pearson's *r*), indicating how they were calculated

Our web collection on statistics for biologists contains articles on many of the points above.

Software and code

Policy information about availability of computer code

Data collection	Excel 2016 (Microsoft Corp., Redmond, WA)
Data analysis	All the statistical analyses were carried out using R programming language version 3.6.0

For manuscripts utilizing custom algorithms or software that are central to the research but not yet described in published literature, software must be made available to editors and reviewers. We strongly encourage code deposition in a community repository (e.g. GitHub). See the Nature Research guidelines for submitting code & software for further information.

Data

Policy information about availability of data

All manuscripts must include a data availability statement. This statement should provide the following information, where applicable:

- Accession codes, unique identifiers, or web links for publicly available datasets
- A list of figures that have associated raw data
- A description of any restrictions on data availability

Source data for Figures 1-3 and for Supplementary Figures 1 and 2 are available in Supplementary Data 1 and 2. References for bioclimatic and socioeconomic data are provided in Table 1. The animal population data owned by Boehringer-Ingelheim Animal Health can be made available upon request (frederic.beugnet@boehringer-ingelheim.com). The other original (raw) data can be made available upon request to the corresponding author (vito.colella@unimelb.edu.au).

Field-specific reporting

Please select the one below that is the best fit for your research. If you are not sure, read the appropriate sections before making your selection.

☒ Life sciences ☐ Behavioural & social sciences ☐ Ecological, evolutionary & environmental sciences

For a reference copy of the document with all sections, see nature.com/documents/nr-reporting-summary-flat.pdf

Life sciences study design

All studies must disclose on these points even when the disclosure is negative.

Sample size	On a monthly basis, we sampled 10 client-owned dogs and 10 client-owned cats in Taiwan, Indonesia, Malaysia, the Philippines, Singapore, Thailand, and Vietnam and 40 dogs and 40 cats in Mainland China for a target total sample size of 2,640 companion animals. Total number of animals sampled are reported in Table S1.
Data exclusions	1) Animals with no access to outdoor environments; 2) Animals that have received antiparasitic treatment within two weeks
Replication	The selection of the best models was based on the following criteria: 1) minimal AIC; 2) maximal AUC for the ROC curve. Besides, we also carried out a posteriori verification of the models by testing the sensitivity of the parameters to extreme values.
Randomization	This is not relevant to our study because there were no experimental groups. Animals were sampled from each region.
Blinding	Blinding is not relevant to our study because there are no experimental groups.

Reporting for specific materials, systems and methods

We require information from authors about some types of materials, experimental systems and methods used in many studies. Here, indicate whether each material, system or method listed is relevant to your study. If you are not sure if a list item applies to your research, read the appropriate section before selecting a response.

Materials & experimental systems

n/a	Involved in the study	
☒	☐	Antibodies
☒	☐	Eukaryotic cell lines
☒	☐	Palaeontology and archaeology
☐	☒	Animals and other organisms
☒	☐	Human research participants
☒	☐	Clinical data
☒	☐	Dual use research of concern

Methods

n/a	Involved in the study	
☒	☐	ChIP-seq
☒	☐	Flow cytometry
☒	☐	MRI-based neuroimaging

Animals and other organisms

Policy information about studies involving animals; ARRIVE guidelines recommended for reporting animal research

Laboratory animals	This study did no involve laboratory animals.
Wild animals	This study did not involve wild animals.
Field-collected samples	All the animals involved in this study were referred to local animal health service centres of the veterinary educational institutes for routine procedures, e.g., pre-surgery, or vaccination.
Ethics oversight	The Ethics Committee of the Department of Veterinary Medicine, University of Bari approved the protocol of this study (protocol no. 13/17).

Note that full information on the approval of the study protocol must also be provided in the manuscript.
